# Profiles of COVID-19 clinical trials in the Chinese Clinical Trial Registry

**DOI:** 10.1080/22221751.2020.1791736

**Published:** 2020-07-22

**Authors:** Peng Xu, Xiangyu Xing, Keying Yu, Zhiguo Lv, Huijing Cui, Yuhang Shi, Tianying Chang, Dongmei Zhang, Yibin Zhang, Kai Wang, Jing Lu, Qingxia Huang, Xiangyan Li, Yingzi Cui, Li Shi, Tan Wang, Junqi Niu, Jian Wang

**Affiliations:** a Neurology Department, The Aﬃliated Hospital to Changchun University of Chinese Medicine, Changchun, People’s Republic of China; b Department of Hepatology, The First Hospital of Jilin University, Changchun, People’s Republic of China; c Editorial Department of Journal of Clinical Hepatology, Changchun, People’s Republic of China; d Cardiovascular Medicine Department, The Aﬃliated Hospital to Changchun University of Chinese Medicine, Changchun, People’s Republic of China; e Traditional Chinese Medicine Department, Nanguan District Traditional Chinese Medicine Hospital, Changchun, People’s Republic of China; f Department of Gerontology, The Changchun Hospital of TCM, Changchun, People’s Republic of China; g GCP Department, The Affiliated Hospital to Changchun University of Chinese Medicine, Changchun, People’s Republic of China; h Scientific Research Office, The Affiliated Hospital to Changchun University of Chinese Medicine, Changchun, People’s Republic of China; i College of Traditional Chinese Medicine, Changchun University of Chinese Medicine, Changchun, People’s Republic of China; j Research Center of Traditional Chinese Medicine, The Affiliated Hospital to Changchun University of Chinese Medicine, Changchun, People’s Republic of China; k Jilin Ginseng Academy, Jilin Provincial Key Laboratory of BioMacromolecules of Chinese Medicine, Changchun University of Chinese Medicine, Changchun, People’s Republic of China; l Respiratory Medicine Department, The Aﬃliated Hospital to Changchun University of Chinese Medicine, Changchun, People’s Republic of China

**Keywords:** COVID-19, anti-viral drug, Traditional Chinese Medicine, clinical trials, vaccine

## Abstract

The COVID-19 pandemic has caused a global public health crisis. There is a pressing need for evidence-based interventions to address the devastating clinical and public health effects of the COVID-19 pandemic. The Chinese scientists supported by private and government resources have adopted extensive efforts to identify effective drugs against the virus. To date, a large number of clinical trials addressing various aspects of COVID19 have been registered in the Chinese Clinical Trial Registry (ChiCTR), including more than 200 interventional studies. Under such an urgent circumstance, the scope and quality of these clinical studies vary significantly. Hence, this review aims to make a comprehensive analysis on the profiles of COVID-19 clinical trials registered in the ChiCTR, including a wide range of characteristics. Our findings will provide a useful summary on these clinical studies since most of these studies will encounter major challenges from the design to completion. It will be a long road for the outcomes of these studies to be published and international collaboration will help the ultimate goals of developing new vaccines and anti-viral drugs.

## Introduction

In December 2019, an unprecedented outbreak of pneumonia cases of unknown aetiology was identified in Wuhan City, Hubei Province, China [1]. A novel coronavirus was identified as the causative agent [2] and was subsequently termed COVID-19 by the World Health Organization (WHO) [3]. Considering its worldwide reach and severity, COVID-19 has been declared a pandemic by the WHO on March 11, 2020, and continues to spread across the globe within about three months. As of May 14, 2020, the COVID-19 outbreak has resulted in 4,248,389 confirmed cases and 294,046 confirmed deaths worldwide [4].

COVID-19 is caused by the infection of severe acute respiratory syndrome coronavirus-2 (SARS-CoV-2), which potently elicits pulmonary hyper-inflammation and infiltration, as well as potentially life-threatening “cytokine storms” [5]. Currently, no vaccines and specific therapeutic drugs are targeting this virus [6]. Most recommendations on preventing disease transmission and treating infected patients are based on anecdotal evidence and the opinions of clinical experts. Supportive management remains the pivot of treatment protocols in the absence of evidence on efficacious anti-viral or anti-inflammatory medications [7]. Although several approved drugs such as hydroxychloroquine and investigational agents such as remdesivir have shown anti-viral activity against SARS-CoV-2 *in vitro* [8,9], a meaningful benefit of these drugs for patients with COVID-19 is urgently needed to evaluate in the clinical studies. To rapidly response the emergent pandemic, the search for an effective and safe treatment regimen has been a major clinical challenge.

During the early stage of the outbreak, the Chinese government has adopted extensive efforts to implement non-pharmaceutical interventions, including inter-city travel restrictions, early identification and isolation of cases, and contact restrictions and social distancing, which have obviously prevented the spread and reduced the outbreak size of COVID-19 across China [10,11].

Importantly, an overwhelming number of clinical trials have been registered in Chinese Clinical Trial Register (ChiCTR) and conducted a comprehensive investigation for a variety of preventive and therapeutic strategies against COVID-19 in patients. ChiCTR is sponsored and established by the Ministry of Health of China in June 2007, which is accepted as the fourth the World Health Organization International Clinical Trial Registry Platform (WHO ICTRP) Primary Register. The profile of COVID-19 clinical trials registered in ChiCTR might reflect the current situation of clinical studies in China, which has not been reported until now. Therefore, this study aimed to describe the characteristics of the currently registered clinical trials related to COVID-19, such as investigational therapies, sponsorship, critical design elements, and specified outcomes. Our analysis could provide timely important information of COVID-19 clinical trials from ChiCTR for clinicians, virus researchers, policy makers, and the general public globally.

## Methods

### Date source

We used the ChiCTR database to identify all COVID-19 clinical trials and retrieve related clinical information on April 24, 2020. A downloadable CSV-type file from the ChiCTR is available, which includes all COVID-19 registered trials and is updating on a daily basis (URL: http://www.chictr.org.cn/index.aspx). Five independent investigators (Huijing Cui, Kai Wang, Yuhang Shi, Li Shi, and Tan Wang) independently conducted a further search for each trial from the ChiCTR by using registration number to obtain the complete trial information from January 23 to April 24, 2020.

### Data extraction

Six investigators (Peng Xu, Zhiguo Lv, Dongmei Zhang, Yibin Zhang, Xiangyu Xing, and Keying Yu) extracted the data of COVID-19 clinical trials and entered them in Microsoft Excel 2010 software. Each record of COVID-19 clinical trials contains several mandatory elements, including general characteristics (ethics approval, clinical trial phase, facilities, enrolment, lead sponsor, overall status), and the design data (the primary purpose, type of intervention allocation, blinding, type of participants, primary outcomes), and candidate interventions (anti-viral drugs, stem cells, and immunomodulators). Two investigators (Qingxia Huang and Jing Lu) used the source register unique identifier to search for peer-reviewed publications (on PubMed and Embase) and pre-print articles (on medRxiv, https://www.medrxiv.org/), which were independently confirmed their correlation to the eligible trials by two investigators (Tianying Chang, and Yingzi Cui). Any discrepancies were resolved by consensus from a panel of investigators within the review team (Xiangyan Li, Junqi Niu, and Jian Wang).

### Data synthesis and analysis

Descriptive statistics are used to characterize the clinical trials. The categorical data are expressed by calculating the frequency and the percentage. All statistical analyses were performed using the SAS software (version9.3; SAS Institute Inc., Cary, NC, USA).

## Results

**Figure 1.**
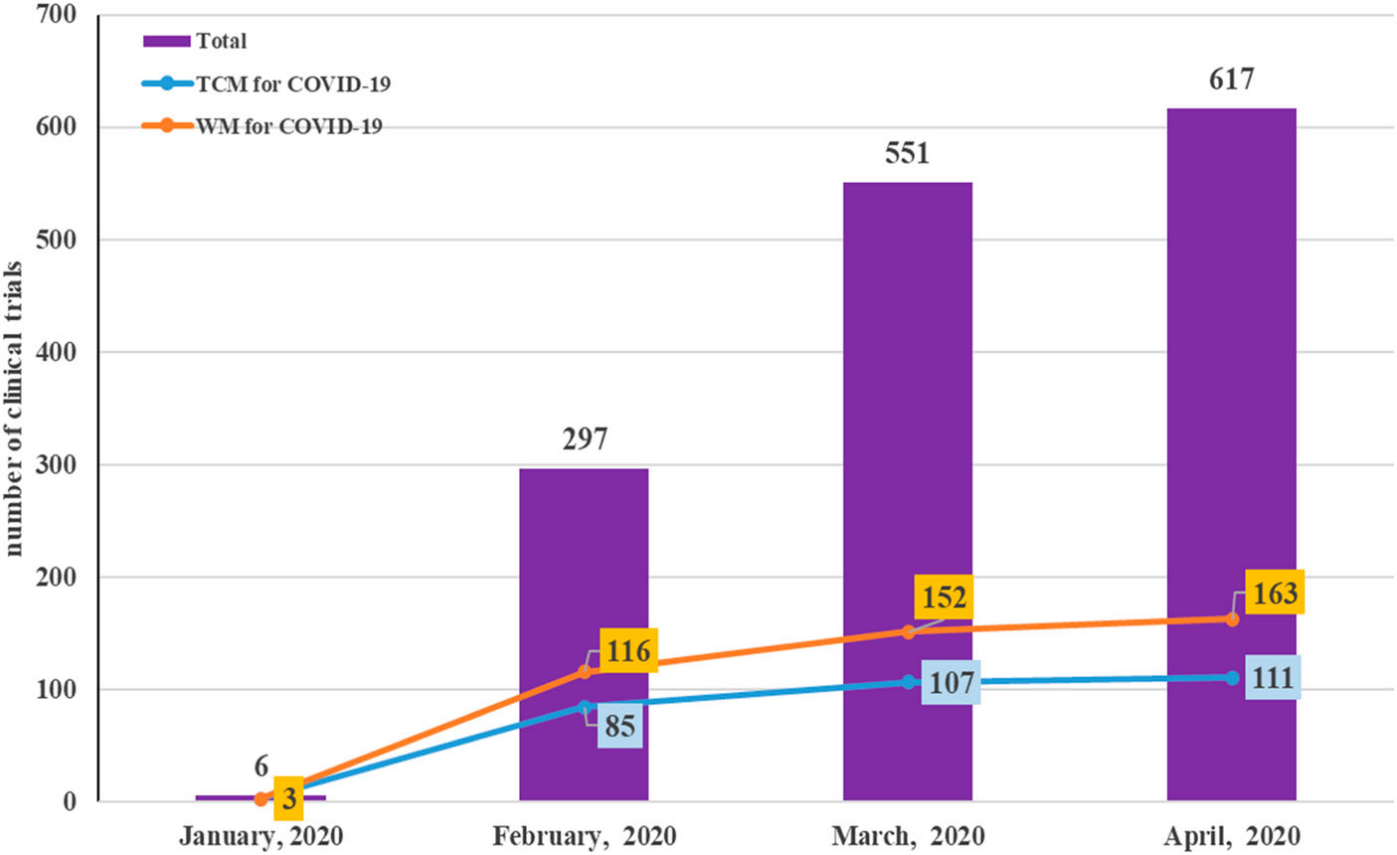
The number of registered COVID-19 clinical trials, ranging from January 2020 to April 2020 in China. The *X*-axis is the trend of the registration date. The Y-axis is the number of COVID-19 clinical trials, and divided into two types of interventions. TCM: traditional Chinese medicine. WM: western medicine.

A total of 617 COVID-19 clinical trials were registered during the period from January 23 to April 24, 2020 and retrieved from the ChiCTR database. The number of these trial registrations increased rapidly during the first three months, and then increased gradually from March to April, especially reaching to a peak in the last one week of April. During these trials, the interventions against COVID-19 included anti-malarial drugs, anti-viral drugs, device (extracorporeal membrane oxygenation, and bronchoscopic alveolar lavage), Taichi behavioural intervention, and Traditional Chinese Medicine (TCM). Impressively, herbal medicine and Chinese patent medicine based on previous clinical evidence and similar public health emergencies such as SARS and H1N1 influenza in China were extensively used and played very important roles in the prevention and treatment of patients with COVID-19 [12]. Compared to TCM intervention, the interventions against COVID-19 such as hydroxychloroquine, remdesivir, vaccine, and stem cells were considered as Western Medicine (WM). We found that the number of clinical trials in both TCM and WM was consistently increased from January to April 2020. Of the 617 COVID-19 clinical trials, 163 trials focused on WM and 111 trials were for TCM. The cumulative numbers of registered COVID-19 clinical trials and the interventions with TCM or WM from January to April in China are shown in Figure 1.

### General characteristics of COVID-19 clinical trials

Table 1 summarizes the general characteristics of COVID-19 clinical trials from the ChiCTR. Of 617 included trials, most trials (521, 84.4%) provided their ethic approvals to the registration system, while 96 trials (15.6%) did not obtain ethics approval. Among those COVID-19 clinical trials, most trials were treatment evaluation (265, 42.9%) as common study purpose, and others focused on the prevention, diagnosis, prognosis, and observational analysis. In the following analysis, we mainly focus on 265 interventional clinical trials of COVID-19 to summary the profile of clinical characteristics. Among 265 interventional trials, 108 trials (40.8%) were phase IV. Phases II and III trials together accounted for 22 trials (8.3%), 6 trials (2.2%) were phase I, and 115 (43.4%) recorded the phase as not applicable. For the enrolment of patients with COVID-19, most trials (121, 45.7%) indicated the enrolment of 100 participants or fewer and 25 (9.5%) had 500 participants or more. A higher proportion of COVID-19 trials (176, 66.4%) conducted the study protocol at a single centre. The most common sponsors of COVID-19 trials were the universities/hospitals (226, 85.3%), and followed by industry (39, 14.7%). In terms of current study status, most of the trials were still recruiting at the time of the analysis (131, 49.4%), followed by 105 trials (39.6%) that were not yet recruiting, and 27 trials (10.2%) was suspended because of lacking patients. Only two trials were completed and the results have been submitted in the platform of ChiCTR.

**Table 1.** General characteristic of COVID-19 clinical trials from ChiCTR.

Variables	No (%)
Ethics approval (*n *= 617)	
Yes	521 (84.4)
No	96 (15.6)
The primary purpose (*n *= 617)	
Treatment	265 (42.9)
Prevention	93 (15.1)
Other purposes	259 (42.0)
Clinical trial phase (interventional studies, *n *= 265)	
Phase I	6 (2.2)
Phase I/ phase II	14 (5.3)
Phase II	15 (5.7)
Phase II / phase III	3 (1.1)
Phase III	4 (1.5)
Phase IV	108 (40.8)
Not applicable	115 (43.4)
Enrolment (interventional studies, *n *= 265)	
0–99	121 (45.7)
100–499	114 (43.0)
500–999	14 (5.3)
≥1000	11 (4.2)
Not available	5 (1.8)
Number of facilities (interventional studies, *n *= 265)	
Single facility	176 (66.4)
2–5 facilities	72 (27.2)
6–10 facilities	15 (5.6)
>10 facilities	2 (0.8)
Lead sponsor (interventional studies, *n *= 265)	
Universities/hospitals	226 (85.3)
Industry	39 (14.7)
Overall status (interventional studies, *n *= 265)	
Recruiting	131 (49.4)
Not yet recruiting	105 (39.6)
Suspended	27 (10.2)
Completed	2 (0.8)

### Design of COVID-19 clinical trials

In 265 interventional trials, the most common interventional model was a parallel design (157, 59.2%). Among those using randomization, the majority used open-label design (247, 93.2%), followed by double-blinded (12, 4.5%), and single-blinded (5, 1.9%) studies. Within all registered 265 COVID-19 interventional trials, a more significant percentage of trials focused explicitly on confirmed cases (78, 29.4%), who were eligible patients with positive RT–PCR result for SARS-CoV-2 and pneumonia confirmed by chest imaging. The top 3 disease conditions of 265 trials were the common case (31, 11.7%), wild case/common case (30, 11.3%), and wild case/severe case (24, 9.1%). Only five trials (1.9%) focused on healthy people. A total of 175 (66.0%) trials chose 1 primary outcome, including the improvement in clinical symptoms, negative nucleic acid detection, or survival. And 90 (34.0%) trials selected more than 1 primary outcome. Table 2 presented detailed information of 265 interventional trials with COIVD-19.

**Table 2.** Design of 265 interventional COVID-19 clinical trials.

Variables	No (%) (*n *= 265)
Allocation	
Randomized	157 (59.2)
Non-randomized	108 (40.8)
Blinding	
Open-label	247 (93.2)
Single-blind	5 (1.9)
Double-blind	12 (4.5)
Triple-blind	1 (0.4)
Type of participants	
Confirmed case	78 (29.4)
Common case	31 (11.7)
Wild case /common case	30 (11.3)
Wild case /severe case	24 (9.1)
Severe case	19 (7.2)
Severe case /critical case	18 (6.8)
Common/severe case	16 (6.0)
Rehabilitation case	11(4.2)
Critical/suspected case	9 (3.4)
Wild case	9 (3.4)
Suspected/confirmed case	8 (3.0)
Suspected case	7 (2.6)
Healthy people	5 (1.9)
Number of primary outcomes	
1	175 (66.0)
2–5	73 (27.5)
6–9	15 (5.7)
>10	2 (0.8)

### Description of candidate interventions in 265 interventional COVID-19 clinical trials

Current therapies for COVID-19 can be divided into two categories, one that can directly target the virus replication cycle such as remdesivir, and the other based on immunotherapy approaches [13,14]. Based on intervention targeting, we found that 115 of 265 trials contained anti-malarial drugs, anti-viral drugs, stem cells, monoclonal antibody, and vaccine, which either aimed to boost innate anti-viral immune responses or alleviated the damage induced by dysregulated inflammatory responses. Of the 256 eligible interventional trials, the most frequently observed intervention was anti-malarial drugs (43, 16.2%). The top three subcategories of anti-malarial drugs were chloroquine phosphate, hydroxychloroquine, and phosphoric chloroquine. The next observed interventions by order of frequency were anti-viral drugs (29, 10.9%), including ritonavir plus lopinavir, favipiravir, azzedine, and baloxavir marboxil, followed by stem cells (16, 6.0%), including mesenchymal stem cells and umbilical cord blood mononuclear cells. Among the immunomodulators, convalescent plasma was the widest intervention (11, 4.2%). Meanwhile, six trials (2.3%) were conducted using monoclonal antibodies, including tocilizumab, adalimumab, ixekizumab, camrelizumab, and PD-1, and followed by corticosteroid (4, 1.5%). In addition, only four clinical trials (1.5%) focused on investigating the efficacy of the vaccine, such as recombinant adenoviral vector, inactivated vero cells or dendritic cell (DC) vaccines. The overview of candidate interventions from 265 COVID-19 trials is presented in Table 3.

**Table 3.** Overview of candidate interventions from 265 COVID-19 clinical trials

Intervention Categories	Intervention Subcategories	No (%) (*n *= 265)
Anti-malarial drugs		43 (16.2)
	Chloroquine phosphate	30
	Hydroxychloroquine	7
	Phosphoric chloroquine	4
	Chloroquine	2
Anti-viral drugs		29 (10.9)
	Ritonavir plus lopinavir	8
	Recombinant interferon	4
	Favipiravir	4
	Azzedine	3
	Baloxavir marboxil	2
	Darunavir	2
	Ganovo	2
	ASC09/ritonavir	1
	Arbidol hydrochloride	1
	Carrimycin	1
	Triazavirin	1
Stem cells		16 (6.0)
	Mesenchymal stem cells	9
	Umbilical cord mesenchymal stem cell-conditioned medium	1
	Umbilical cord blood mononuclear cells	1
	Menstrual blood-derived stem cells	1
	Human menstrual blood-derived stem cells	1
	NK cells and mesenchymal stem cells	1
	Umbilical cord blood plasma	1
	Cord blood mesenchymal stem cells	1
Immunomodulators		13 (4.9)
	Convalescent plasma	11
	CMAB806 (IL-6)	1
	NLRP Inflammasome inhibitor (Tranilast)	1
Monoclonal antibodies		6 (2.3)
	Tocilizumab	2
	Adalimumab	1
	Ixekizumab	1
	Camrelizumab	1
	PD-1	1
Corticosteroid	Methylprednisolone	4 (1.5)
Vaccine		4 (1.5)
	Vaccine (adenoviral vector)	2
	Vaccine (vero cells)	1
	DC vaccine	1

## Discussion

To our knowledge, this study provides an extensive and complete descriptive assessment of COVID-19 trials registered in the ChiCTR. According to the information, we have collected, our results show that the clinical trial researches primarily assess whether a wide range of existing therapies might be effective against COVID-19. Among of these clinical trials, more than one half are open label and of limited sample sizes with enrolling a median (IQR) of 120 (IQR 67–300) patients, which are likely to evaluate preliminary evidence of the effectiveness against COVID-19.

Our results showed that a few trials did not obtain ethics approval, in which the patients may become more vulnerable to potential coercion, exploitation, and experience limited mental capacity to make informed choices [15]. For the future clinical research for COVID-19, ethics approval is essential for assessing the potential benefit of the participants. We found that the sample size of registered and ongoing COVID-19 trials was small and limited, which is hard to conduct secondary and subgroup analyses. For example, only 200-patient randomized controlled trial showed that lopinavir plus ritonavir had no treatment benefit beyond standard care against COVID-19, which may be related with limited cases as mentioned by the authors [16]. Moreover, the overall status of COVID-19 trials showed that a proportionately larger number remained recruiting and not yet recruiting. Like Ebola, the COVID-19 epidemic was waning at the onset of the trials, making it impossible to enrol enough cases for reaching definitive endpoints [17]. For instance, two trials for remdesivir in China (Clinical Trial Number: NCT04252664 and NCT04257656) were halted due to a lack of patients with COVID-19. Therefore, we recommend that some trials from ChiCTR might not be able to enrol enough patients up to their pre-established sample size. However, imported or asymptomatic infection cases may cause the second wave of COVID-19 epidemic in China, it is possible to recruit more cases to satisfy the design of sample size. Some trials used the single-arm design without comparison with a control group. As we have known, single-arm studies demonstrate the benefit of a new treatment, which generally used an external reference comparator [18]. For a novel emerging acute respiratory infectious disease, it was unable to gather any reliable, comparable, and historical data of the interventions, which will be a big challenge for evaluating exactly therapeutic efficacy of the intervention against COVID-19. In addition, primary outcomes can be used to define meaningful evidence for the results of an intervention [19]. In our analysis, most trials chose surrogate outcomes such as time to clinical recovery instead of the mortality, which is easy to evaluate the clinically meaningful effect of the intervention under investigation in smaller sample size and shorter duration, thus accelerate the approval for clinical application. Overall, it is necessary to make standard and reasonable design for COVID-19 clinical trials before the starting.

When compared to other databases, like ClinicalTrials.gov, our results indicate that early registered trials focus on the interventions with TCM, which is associated with the COVID-19 outbreak started in China. TCM have been repurposed for COVID-19 treatment as recommended by the National Health Commission of China (NHCC). On April 14, 2020, the National Medical Products Administration approved three Chinese medicines, including Xuebijing Injection, Jinhua Qinggan Granule, and Lianhua Qingwen Capsule for authorization in the treatment of COVID-19 patients, albeit without clear mechanistic knowledge and little clinical validation [20]. The first multicenter, prospective, and randomized controlled trial showed that Lianhua Qingwen Capsule could ameliorate clinical symptoms of COVID-19 in light of the safety and effectiveness profiles [21]. Importantly, an extensively explored strategy against COVID-19 is drug repurposing. Based on previous evidences from SARS and MERS WHO guidelines do not recommend corticosteroids to treat the patients with COVID-19, because corticosteroids do not reduce mortality and potentially delay viral clearance [22]. Hydroxychloroquine, which may act by increasing the pH within lysosomes, was granted FDA authorization for emergency cases [23]. However, current results demonstrate that hydroxychloroquine [24] and lopinavir plus ritonavir [25] have no benefit for COVID-19 patients in randomized trials. Several trials of remdesivir treatment on a few patients in the United States have shown early promising benefits in cases with severe pneumonia [26,27]. A phase III clinical trial of remdesivir for COVID-19 launched by Chinese health authorities found that the treatment with remdesivir did not shorten the duration of illness or reduce the mortality of hospitalized COVID-19 patients, compared with the placebo [28]. We think that this difference in the results of remedsivir clinical trials might be related with different primary outcomes, which should be further investigated in large and standardized trials. Once the outbreak is under control locally, there might not be sufficient patients to be enrolled for ongoing studies in the region. In the post-COVID-19 era, vaccine development is of the utmost priority. At present, there are no approved vaccines for SARS-CoV-2, a growing number of global public and private institutions have joined the efforts to develop vaccines against SARS-CoV-2 [29]. As of May 15, the WHO has filed 110 candidate vaccines in the preclinical evaluation and eight candidate vaccines in clinical evaluation [30]. Among eight clinical researches, four candidate vaccine trials were performed in China, three were in the United States, and one was in the United Kingdom. Non-replicating viral vectors, RNA, DNA, and inactivated vaccines were evaluated and developed to prevent the COVID-19 epidemic [31].

Our study has some limitations. Our analysis was a cross-sectional summary of trials registered in the ChiCTR in the early pandemic of COVID-19, which did not cover the updating clinical trials in this platform. We mainly focused on the analysis of the interventional trials against COVID-19, not for observational trials on the diagnosis and prognosis of patients with COVID-19. In addition, the data of this analysis was just from the ChiCTR.

Based on our findings and current status of COVID-19 epidemic, four key points need to been concerned: (1) Investigators from the globe should be more collaborative and join their efforts in the conducting multicenter trials to test the efficacy of the intervention, (2) The well-public and network platform should be established monitor the quality and necessity of design proposal for clinical trials, (3) Non-pharmaceutical interventions such as hand hygiene, masks, and early case identification and isolation should be maintained to prevent and delay the arrival of the second wave of COVID-19 epidemic.

## Conclusion

Here, this review summarizes COVID-19 clinical trials registered in the ChiCTR in the early outbreak of COVID-19 pandemic. Most of these studies will encounter major challenges from the design to completion, which has a long road for the outcomes of these studies to be published. International collaboration will help the ultimate goals of developing new vaccines and anti-viral drugs.
